# Non-surgical treatment of hidradenitis suppurativa: the role of cryotherapy

**DOI:** 10.3389/fmed.2023.1141691

**Published:** 2023-04-18

**Authors:** Massimo Dell'Antonia, Jasmine Anedda, Alice Tatti, Alessandro Falco, Silvia Sanna, Caterina Ferreli, Laura Atzori

**Affiliations:** Dermatology Clinic, Department of Medical Sciences and Public Health, University of Cagliari, Cagliari, Italy

**Keywords:** hidradenitis suppurativa, cryotherapy, cryosurgery, cryoablation, non-surgical treatment, hidradenitis suppurativa—acne inversa—therapy, hidradenitis suppurativa—ultrasonography, hidradenitis suppurativa (HS)

## Abstract

**Background:**

Hidradenitis suppurativa is a chronic and recurrent inflammatory disease with a great impact on a patient's quality of life, due to the painful involvement of very sensitive areas, such as the groin, mammary area, and genitals, with malodourous discharge. Multiple treatment options are available; however, no single treatment is effective for all patients, and usually, a combination of medical therapy with various surgical and physical procedures is provided. Cryotherapy is not a routine technique to treat HS, although usually available in the majority of medical clinics, and is cheaper than laser and surgical treatments. The aim of this study was to evaluate the effectiveness of cryotherapy on persistent HS nodules, to reduce the burden of local disease.

**Materials and methods:**

Retrospective observational study in all patients treated with liquid nitrogen cryotherapy for persistent nodules of hidradenitis suppurativa in the last 2 years, with at least 6 months of follow-up after the procedure. Disease severity was assessed with Hurley staging and sonographic staging according to SOS-HS (18 MHz probe, Esaote-MyLab™). The results were scored with a 0–3 points scale, as complete remission (3), partial response (2 to 1), or no response (0), after one session of treatment. Local cleansing and antiseptic treatment after the procedure was the same as previously performed in each patient, not to influence recovery.

**Results:**

In total, 23 patients were included, with a total of 71 persistent nodules treated with a single cryotherapy session. The treatment has been effective in 63 out of 71 nodules treated (88.7%), and the patients attested that they recommend the treatment, the discomfort during recovery was minimal, and the management was not different from daily routine. Persistence was considered as having a failure rate (11.3% overall) and occurred in 7.5% of the nodules of the axillary region, 18.2% on the groin, and 11.2% for nodules of the gluteal region.

**Conclusion:**

Cryotherapy is a simple and effective procedure for the treatment of persistent nodules of HS not responding to medical therapy, and it is a valid alternative to local surgery or laser ablation.

## Introduction

Hidradenitis suppurativa (HS), also known as acne inversa or Verneuil's disease, is a chronic, recurrent inflammatory skin disorder that primarily affects the follicular-pilosebaceous units (1). HS has a variable prevalence estimated at 1–4% of the general population and is more common in young adults and in females (2). The disease predominantly occurs in the apocrine gland-bearing areas of the body, most commonly in the axillary, inguinal, perianal, gluteal, and inframammary regions. The clinical manifestations are variable, including recurrent inflamed nodules, deep abscesses, and draining sinuses and scars (1). HS is characterized by recurrent flares, associated with pain, malodor drainage, and disfigurement, leading to a significant physical, social, and functional impairment, with a negative impact on the quality of life (3, 4).

Treatment of HS is still challenging, often with unsatisfactory results. The main targets are to reduce the formation of new inflammatory lesions, heal existing lesions, and minimize associated symptoms, depending on the clinical stage and severity (5). Recent evidence suggests an inverse correlation between the therapeutic delay and clinical response, supporting a very limited treatment window to control the disease progression and avoid end-stage severe fibrosis and scarring (6). Early treatment, with a quite aggressive approach, is suggested. Among numerous options available to reduce the local burden of the disease, surgical techniques are most commonly used for persistent lesions, not responding to medical therapy. Cryotherapy is not a routine technique to treat HS, and there is a lack of studies about this therapeutic option. Simple external cryotherapy has been evaluated in only one study (7). A few studies evaluated cryoinsufflation, performed by injecting liquid nitrogen through a 21-gauge needle directly into HS tracts, promoting obliteration with scarring (8–10). Nevertheless, this procedure can be associated with some adverse effects such as vagal reaction and an increased risk of air embolism and subcutaneous emphysema.

In our clinic, cryotherapy is often used to treat persistent nodules of HS not responding to topical and systemic treatment, with a general impression of high effectiveness and patient's good compliance. Thus, the idea was to perform a retrospective study, to analyze results on persistent nodules of hidradenitis suppurativa in a sample of well-documented patients, with a follow-up period of 6 months after the single cryotherapy session.

## Materials and methods

We have included in our study all patients treated with cryotherapy for nodules of hidradenitis suppurativa from 1 January 2021 to 31 December 2022, with at least 6 months of follow-up after the procedure. Only patients with isolated persistent nodules, not responding to medical therapy, both topical and systemic, were candidates for this treatment and included in our study. Persistence was defined as no improvement after at least 16 weeks of treatment.

All patients were taking a concomitant systemic therapy that had not changed during the previous 12 weeks. Written informed consent was obtained from all patients. Disease severity was assessed with Hurley staging and sonographic staging according to SOS-HS (18 MHz probe, Esaote-MyLab™). Ultrasound better characterized clinical inflammatory nodular lesions as pseudocystic nodules and/or abscesses, which were all treated.

Exclusion criteria were history of surgery and CO_2_ laser ablation in the same affected regions.

The treatment was performed using liquid nitrogen applied to the lesions using a cryogun (CryoPro; Cortex Technologies ApS, Denmark). Overall, three different and interchangeable operators performed the session in the 2 year-time-lapse, as per common clinical practice. Each lesion was treated with one freeze–thaw cycle of variable duration from 20 to 50 s. As per common clinical practice, the duration of freezing depended on the size and the location of the lesion: Short freeze–thaw cycles of 20–40 s were used for small nodules or nodules on the groin, whereas big nodules or nodules on the axilla required longer freeze–thaw cycles of 40–50 s. All patients were asked to rate the pain of the procedure on a scale of 1–10 and the acceptability in terms of willingness or not (Yes/No) to repeat the treatment. The results for each lesion treated, after one session of cryotherapy, were scored with a 0–3 points scale, on the basis of the volume reduction of the lesion, as complete remission (3: nodule not detectable at follow-up, neither clinically nor ecographically), partial response (2–1; 2: nodule detectable at follow-up but reduced in volume by >50%, 1: nodule reduced in a volume of <50% and or detectable at ultrasound), or no response (0: nodule still present at follow-up without a significant reduction in volume). Medication after treatment did not differ from daily routine, with antiseptic soap and soothing emollients previously in use in each patient, until complete healing of the freezing burns. All patients have been evaluated after 3 months and 6 months.

## Results

Our results are reported in Table 1. We have identified 23 patients, 17 female and 6 male subjects, aged 22–58 years (with a mean age of 34.6 years) treated with cryotherapy for persistent nodules of hidradenitis suppurativa from 1 January 2021 to 31 December 2022, with at least 6 months of follow-up after the procedure. Disease duration ranged from 5 to 23 years (with a mean duration of 12.7 years), eight patients (34.8% of patients) were in the Hurley 1 stage, 11 patients (47.8%) were in the Hurley 2 stage, and four patients (17.4%) were in the Hurley 3 stage. According to SOS-HS, three patients (13.1%) were staged as grade 1, 11 as grade 2 (47.8%), and nine as grade 3 (39.1%).

**Table 1:** Patient details.

**Pt. no**	**Sex**	**Age**	**Duration of HS (years)**	**Sites involved**	**Hurley stage**	**Sonographic score (SOS-HS)**	**Concomitant medical therapy**	**Sites and number of lesions treated**	**Pain during procedure (1-10)**	**3 months follow-up visit: Healing of freezer burns? Persistence of the nodules?**	**6 months follow-up: Persistence of the nodules? Recurrence?**
1	F	37	7	Armpit, groin	2	2	Antibiotic, estroprogestinic pill	Groin (3 nodules)	8	Healing, complete remission of all nodules	No recurrence
2	F	29	13	Armpit, groin, buttocks	2	3	Adalimumab	Armpit (2)	2	Healing, complete remission of all nodules	No recurrence
3	F	38	21	Groin	1	2	Antibiotic	Groin (2)	6	Healing, persistence of 2 nodules of the groin (no response)	Persistence of 2 nodules of the groin (no response)
4	F	28	14	Armpit, breast, groin	2	3	Antibiotic	Armpit (1), groin (1)	5 (armpit), 8 (groin)	Healing, complete remission of all nodules	No recurrence
5	M	39	18	Armpit, groin, buttocks	3	3	Adalimumab	Armpit (4), buttocks (2) (right buttock on Figure 1)	3 (armpit), 2 (buttocks)	Healing, complete remission of all nodules (right buttock on Figure 1)	No recurrence
6	F	34	14	Armpit, groin, buttocks, breast	2	3	Antibiotic, estroprogestinic pill	Armpit (3)	4	Healing. complete remission of all nodules	No recurrence
7	F	38	22	Armpit, groin; buttocks	3	3	Adalimumab	Groin (2) (Figure 2)	7	Healing, complete remission of all nodules (Figure 2)	No recurrence
8	F	33	18	Armpit	2	2	Antibiotic	Armpit (4)	5	Healing, complete remission of all nodules	No recurrence
9	F	22	7	Armpit, groin	1	3	Antibiotic	Armpit (5)	6	Healing, persistence of 2 nodules of the armpit (no response)	Persistence of 2 nodules of the armpit (no response)
10	F	23	5	Armpit	2	2	Antibiotic, estroprogestinic pill	Armpit (2)	4	Healing, complete remission of all nodules	No recurrence
11	M	24	8	Armpit, buttocks	1	2	Antibiotic	Buttocks (4)	5	Healing, persistence of 1 nodule of the buttock (partial response, score 1)	Persistence of 1 nodule of the buttock (partial response, score 1)
12	F	29	14	Armpit, groin, breast	2	3	Antibiotic	Armpit (2) + groin (1) (Figures 3, 4)	4 (armpit), 8 (groin)	Healing, complete remission of all nodules	No recurrence (Figures 3, 4)
13	M	48	13	Armpit, buttocks	2	2	Adalimumab	Armpit (2)	3	Healing, complete remission of all nodules	No recurrence
14	M	32	6	Armpit	2	3	Antibiotic	Armpit (5)	5	Healing, complete remission of all nodules	No recurrence
15	F	24	7	Armpit, groin	1	2	Antibiotic	Armpit (1) + groin (2)	2 (armpit), 7 (groin)	Healing, complete remission of all nodules	No recurrence
16	F	58	9	Armpit, groin	2	2	Adalimumab	Groin (3)	6	Healing, complete remission of all nodules	No recurrence
17	M	43	23	Armpit, groin	3	3	Adalimumab	Armpit (4) + groin (2)	3 (armpit), 8 (groin)	Healing, complete remission of all nodules	No recurrence
18	F	28	21	Armpit, groin, breast, buttoocks	3	3	Adalimumab	Armpit (2) + groin (1) + buttocks (3)	3 (armpit, buttocks), 7 (groin)	Healing, complete remission of all nodules	No recurrence
19	M	31	10	Armpit	2	2	Antibiotic	Armpit (1) (Figure 5)	3	Healing, persistence of 1 nodule of the armpit (partial response, score 2) (Figure 5)	Persistence of 1 nodule of the armpit (partial response, score 2)
20	F	45	15	Groin	1	1	Antibiotic	Groin (1)	3	Healing, persistence of 1 nodule of the groin (no response)	Persistence of 1 nodule of the groin (no response)
21	F	51	13	Armpit, gorin	1	2	Antibiotic	Armpit (2) + groin (1)	5 (armpit), 5 (groin)	Healing, complete remission of all nodules	No recurrence
22	F	39	8	Groin	1	1	Antibiotic	Groin (2)	9	Healing complete remission of all nodules	No recurrence
23	F	23	6	Groin	1	1	Antibiotic, estroprogestinic pill	Groin (1)	5	Healing, persistence of 1 nodule of the groin (no response)	Persistence of 1 nodule of the groin (no response)

Persistence of the nodules at 3 months follow-up was evaluated with a 0–3 points scale, as complete remission (3), partial response (2 to 1), or no response (0), after one session of treatment.

**Figure 1:**
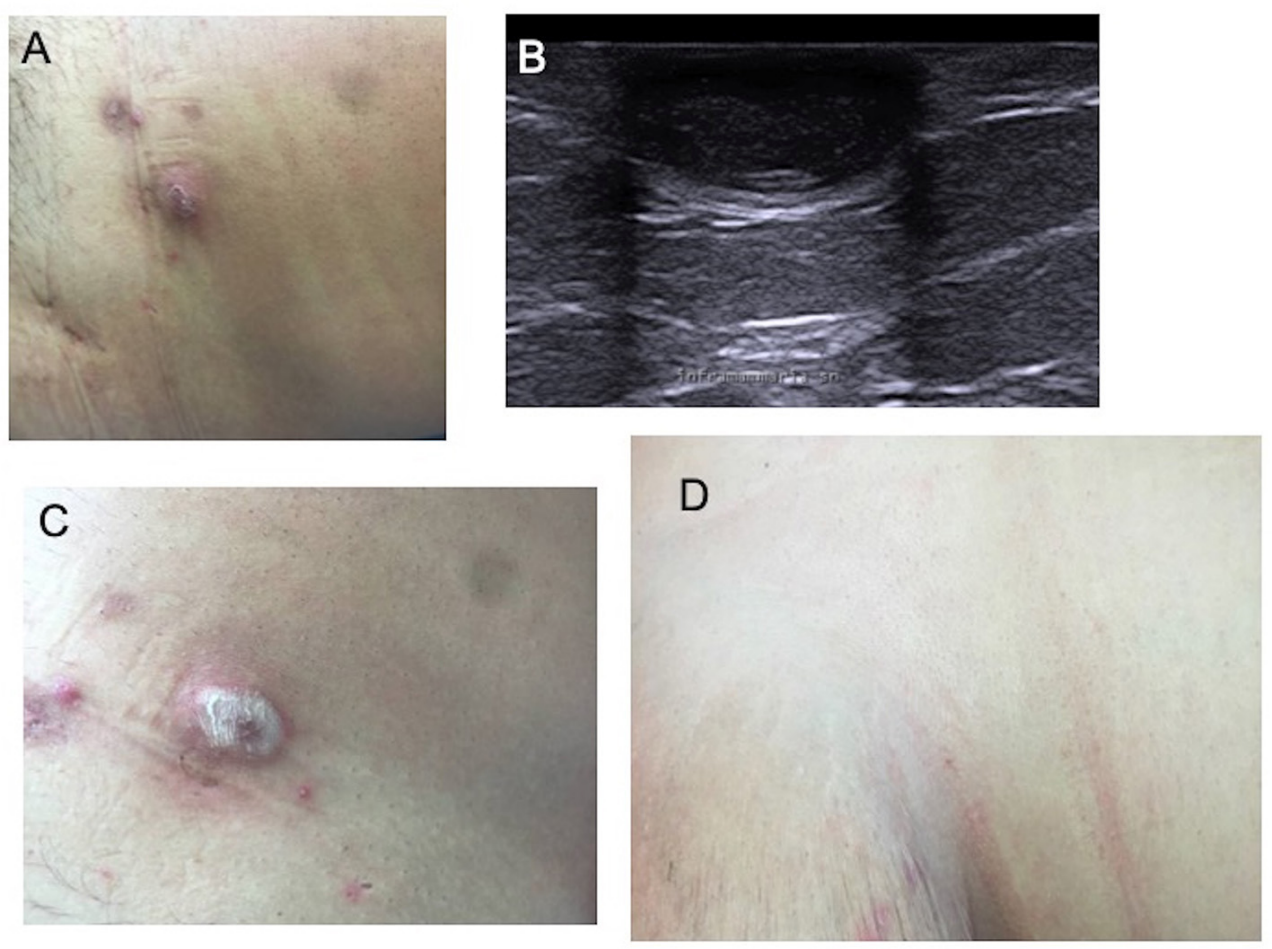
Nodular lesion of hidradenitis suppurativa of the right buttocks **(A)**; ultrasound imaging documenting a roundish hypoechoic nodule in the soft tissue **(B)**; immediately after cryotherapy **(C)**; complete resolution of the lesion at 3-month follow-up **(D)**.

**Figure 2:**
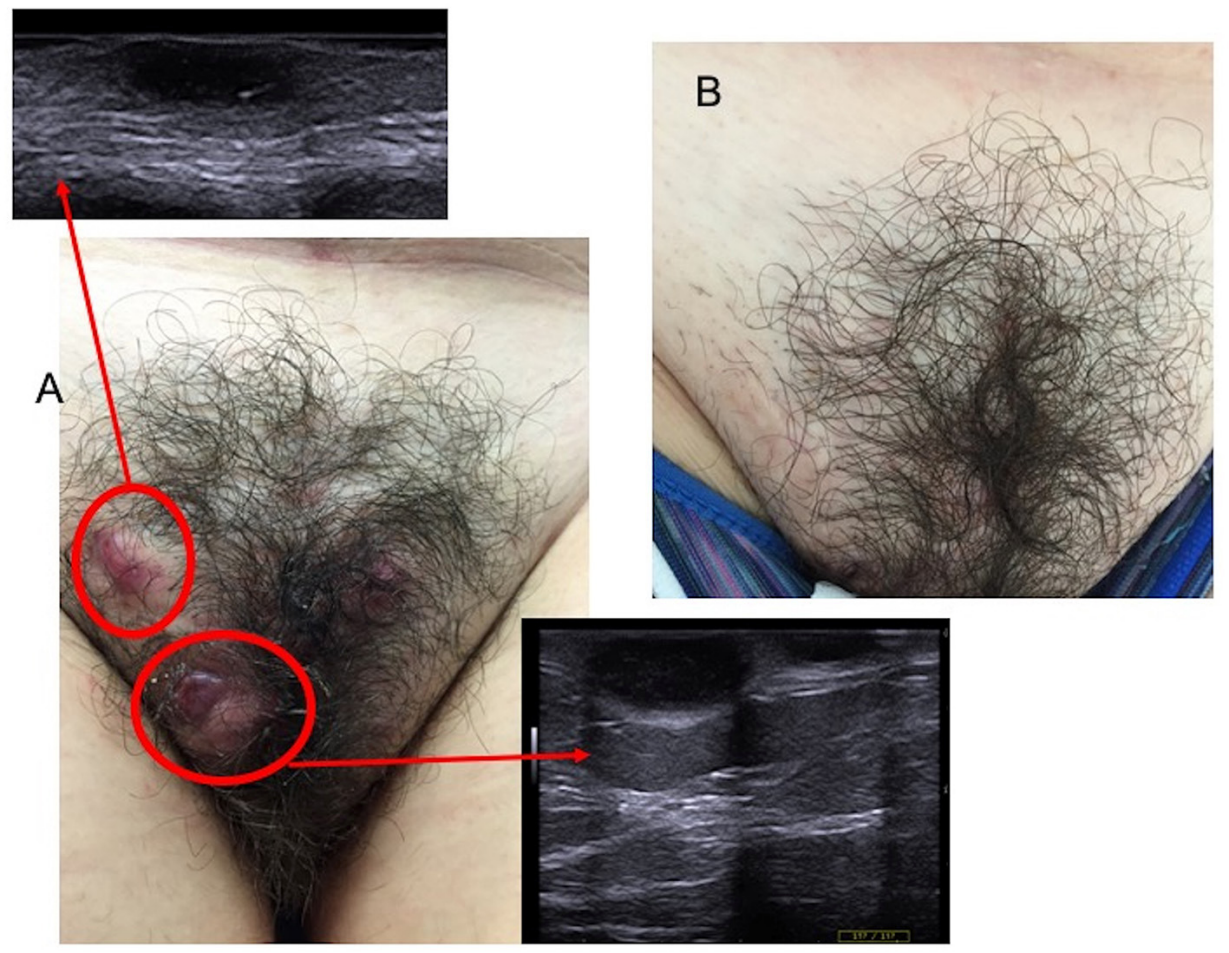
Nodular lesions of the groin with the ultrasound assessment before cryotherapy **(A)**, and complete resolution at 3-month follow-up **(B)**.

**Figure 3:**
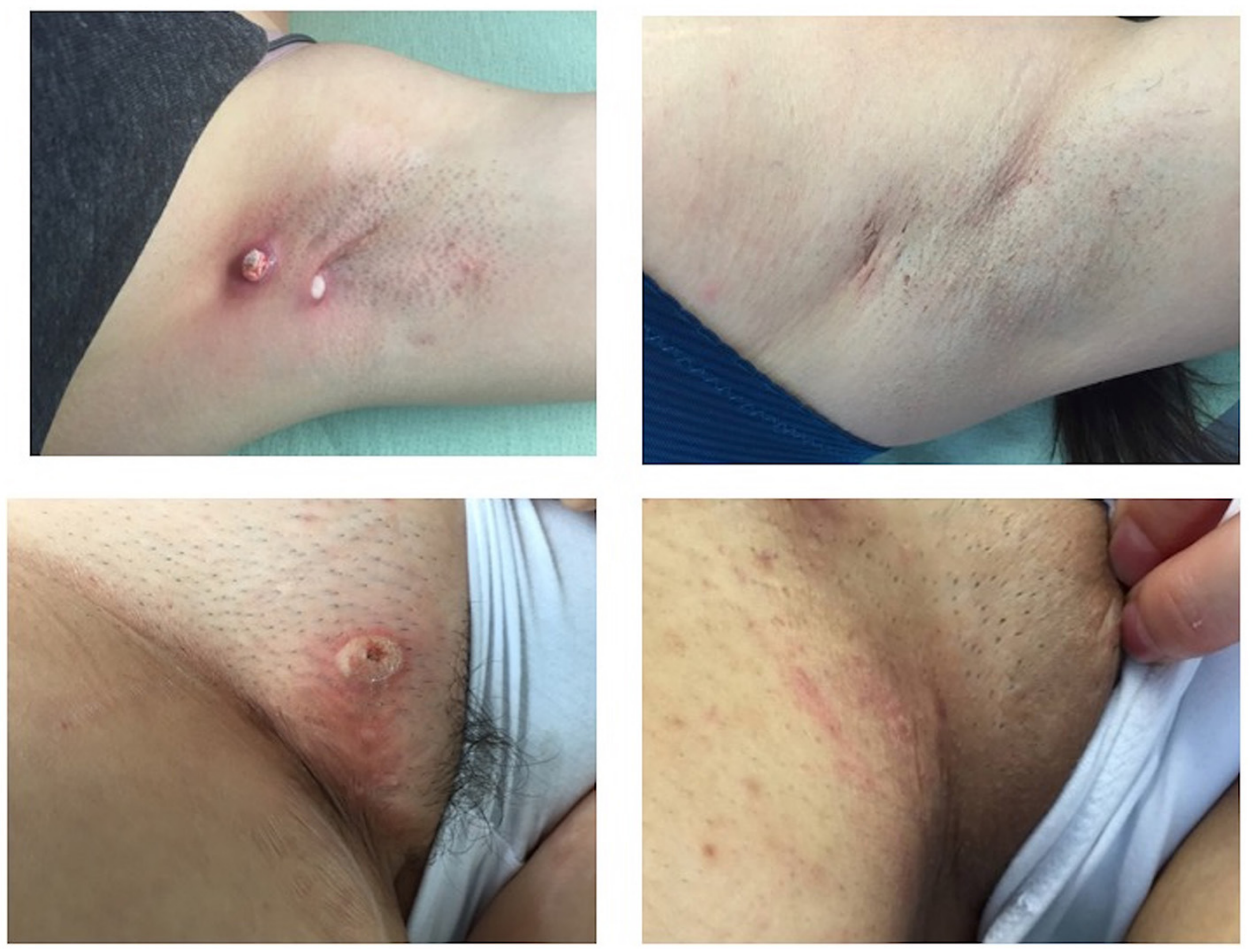
Hidradenitis suppurativa lesions of the armpit and of the groin immediately after cryotherapy and complete resolution at the 6-month follow-up.

**Figure 4:**
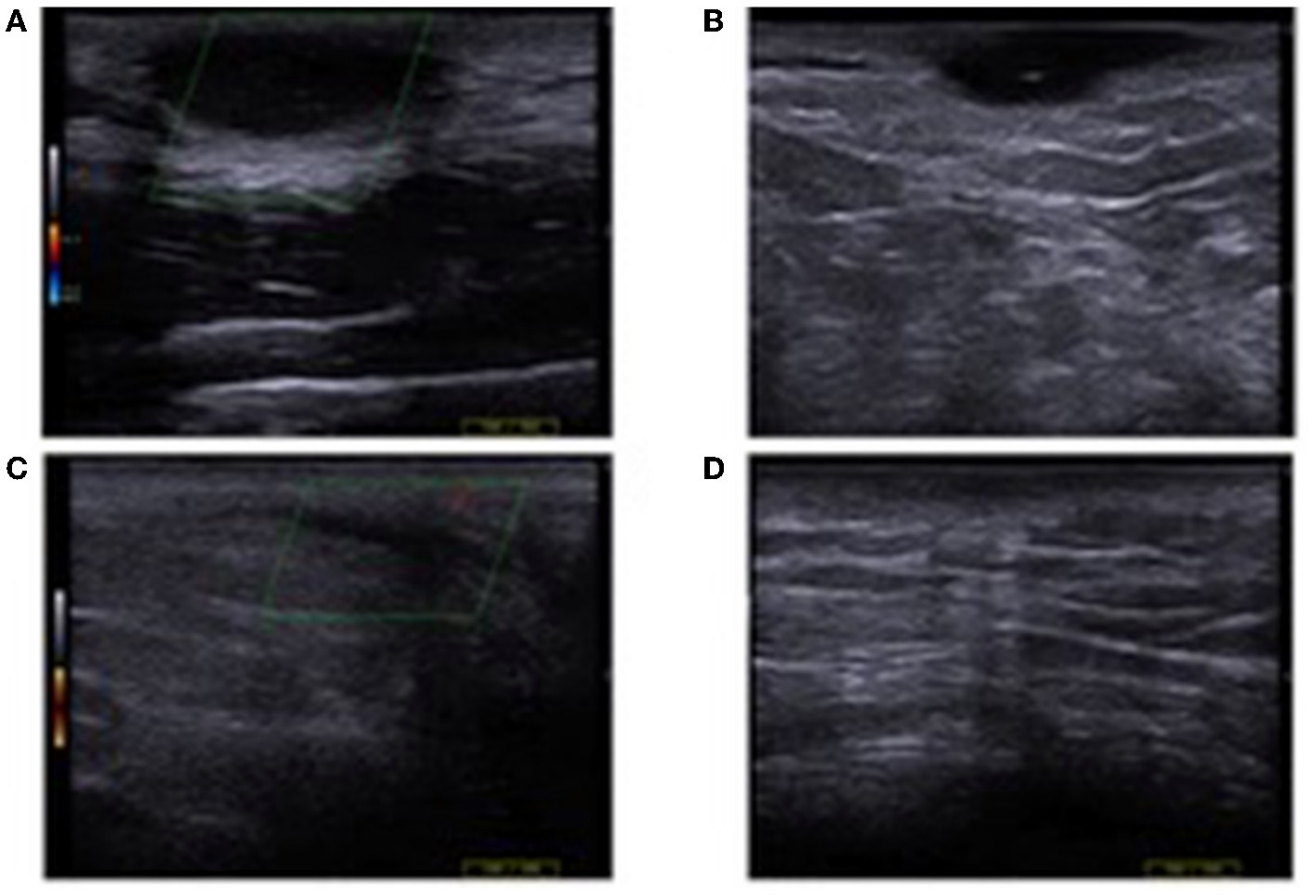
Two pseudo-nodular lesions of the right armpit, before cryosurgery **(A, B)** and complete resolution at 6-month follow-up **(C, D)**.

**Figure 5:**
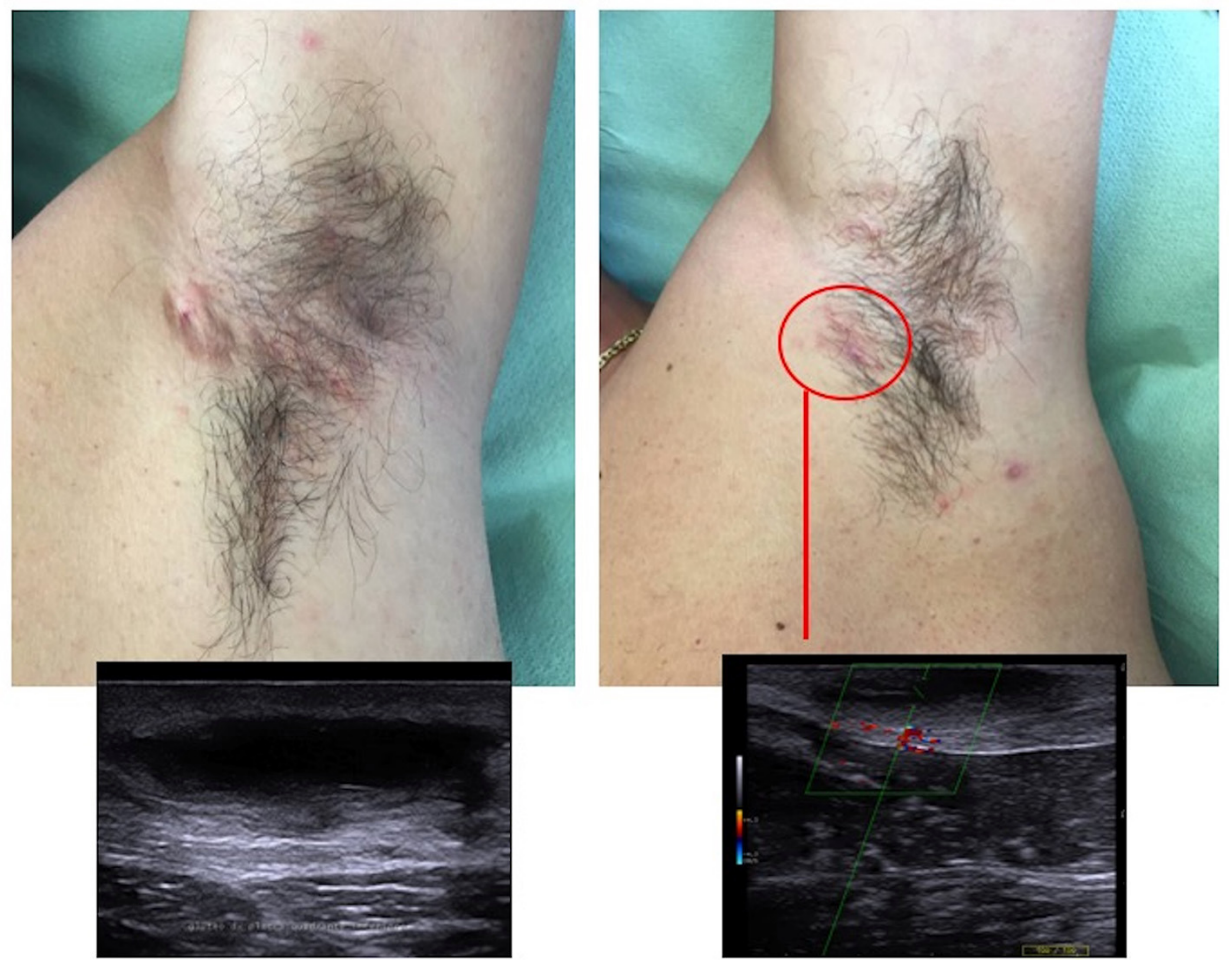
Hidradenitis suppurativa lesions of the armpit before and after cryotherapy, with ultrasound assessment documenting improvement, but the persistence of a pseudocyst hypoechoic nodule with the fibrotic surrounding tissue.

Treated lesions were mainly localized in the axillary region (15 patients; 65.2%), followed by the groin (13 patients; 56.5%) and gluteal region (3 patients, 13%). All patients were taking a concomitant systemic therapy: 16 patients (69.6%) were taking antibiotic therapy, and six patients (30.4%) were treated with adalimumab. A total of four female patients were also taking estro-progestinic pills. The medication schedule was stable and did not change for at least 12 weeks, so as not to interfere with the local treatment evaluation.

A total of 71 persistent nodules have been treated with cryotherapy, from 1 to 7 nodules per patient (mean 3.1 nodules); 40 nodules were localized in the axillary region, 22 nodules in the groin/pubic area, and 9 nodules on the buttocks.

The pain score during the procedure ranged from 2 to 9, with a mean score of 5, with maximum sufferance for the inguinal region, ranging from 3 to 9, with a mean score of 6.7. All patients rated the procedure as acceptable (100%); seven patients considered cryotherapy as better than oral antibiotics, and eight patients would consider this treatment again in the future. No differences in response rate were observed considering the performing physician or site of involvement.

At the 3-month follow-up, all the freezer burns were healed without sequelae, and the patients did not complain of excessive discomfort or complications during healing. A complete response to treatment was clinically and sonographically assessed in 63 out of 71 (88.7%). Only eight lesions persisted (three nodules of the axillary region, four on the groin, and one on the buttocks), six lesions belonged to the “no response” category (score 0), and two lesions belonged to the “partial response” category (respectively score 1 and score 2). At the 6-month follow-up, no recurrence of the regressed nodules was observed, and a second cycle of cryotherapy was performed on the eight persisting lesions, with success.

Overall, the single treatment was effective in 63 out of 71 nodules treated (88.7%): 37 out of 40 nodules of the axillary region (92.5%), 18 out of 22 nodules of the groin (81.8%), and eight out of nine nodules of the gluteal region (88.8%). The overall failure rate was 11.3% (7.5% for nodules of the axillary region, 18.2% for nodules of the groin, and 11.2% for nodules of the gluteal region).

## Discussion

Hidradenitis suppurativa is a chronic and debilitating disease with a great impact on a patient's quality of life. Multiple treatment options are available nowadays, including medical therapy, surgery, and lasers. Despite the various guidelines and the multiple treatment regimens available, the management of the disease is still challenging, and no single treatment has been proven to be effective for all patients (5, 6, 11–14). Thus, it is crucial to have several options to integrate and tailor to each patient.

Cryogun cryotherapy with liquid nitrogen is a cheap and quick minimally invasive technique widely used for the treatment of numerous skin lesions, most commonly benign tumors, but it can be used also in inflammatory conditions, such as papulo-pustular acne (15, 16). To date, in indexed literature, there are only few articles investigating the role of cryotherapy in HS lesions (7–10), mainly supporting cryoinsufflation as an effective technique. It is performed by injecting liquid nitrogen through a 21-gauge needle directly into HS tracts. A case series of 10 patients evaluated a punch-trocar-assisted cryoinsufflation (cryopunch) after the drainage of abscesses and achieved a high percentage of complete resolution, with no recurrence or adverse events at a 6-month follow-up. The main limitation of the study was the small patient's population included (8). However, cryoinsufflation can be associated with severe adverse effects, such as vagal reaction with nausea and sweating and an increased risk of air embolism and subcutaneous emphysema (8, 9). Bong et al. (7) are the only authors considering the role of traditional external cryotherapy in persistent painful nodules of hidradenitis suppurativa, and they found that 8 over 10 patients benefited from this treatment.

In our study, we have evaluated the effectiveness of cryotherapy for the treatment of persistent HS nodules of the axilla, groin, and gluteal region, not responding to at least 16 weeks of systemic medical therapy. Our cohort is composed of 23 HS patients, and the systemic treatment included antibiotics, estro-progestinic pills, and adalimumab. All the lesions treated with cryotherapy were also treated topically without a consistent response.

A complete resolution has been clinically and ecographically documented in 88.7% of the lesions treated after one cryotherapy treatment, and in only 11.3% of the nodules, the treatment was ineffective. No recurrence of the regressed nodules has been identified after 6 months. Ultrasound allowed a better classification of the inflammatory lesions, confirming the crucial role in the assessment of the disease severity (17, 18). However, the abscess showed a favorable response and pseudocystic nodules, and the general appearance of the skin improved in the treated areas. A second cycle of cryotherapy was performed on residual eight lesions, with a complete response.

An advantage of cryotherapy with respect to surgery and cauterization is that cooling triggers a strong intervention of immunologic mechanisms, prolonged vascular stasis, and additional stromal remodeling that enable a very natural wound healing (19, 20). Cryotherapy allows freezing of the lesion in toto, including the fibrotic walls. Although deeper tissues might not be evenly frozen as with cryoinsufflation, the latter causes severe damage and potentially severe adverse events. In our opinion, it is not necessary to produce massive necrosis as even a quote of cellular injury in the area exposed to very low temperatures would produce irrecoverable damage and cell death through apoptosis, which will trigger the immune response. Moreover, osmolarity changes and crystal formation might directly affect the biofilm and bacterial traps that are usually resistant to local and systemic treatment. Finally, recovery after conventional cryotherapy is fast, with minimal downtime or pain.

In fact, the procedure was well tolerated, with a mean pain score of 5; and higher scores were reported for lesions in the inguinal area (with a mean score of 6.7) than axillary and gluteal areas (with a mean score of 3.8 and 3.3, respectively). From our data, it should be considered the use of a topical anesthetic for nodules of the groin, whereas it is not necessary for nodules of the axilla or gluteal region.

It is necessary to inform the patient about the possible complication of cryotherapy, which is usually localized to the site involved, including blistering, edema, pain, and ulceration. Infections are uncommon and prevented by topical antibiotics or antiseptic agents currently used for HS (21). In our cohort, no patient complained of excessive discomfort or complications during healing, and all patients were prescribed to continue their daily routine, with antiseptic soap and an antibiotic ointment to prevent infections.

In summary, in our group of 23 patients with persistent HS nodules, we found that cryotherapy is a useful and effective adjunctive treatment. Early and appropriate interventions are crucial to increase the success rate of the treatment of the disease and prevent the consolidation of resistant HS lesions, such as tunnels and fibrotic scars. Many studies have proved the effectiveness of surgical techniques, such as excision or deroofing (22), and a few studies have determined the benefits of carbon dioxide laser ablation (23); however, these techniques need long recovery times and are quite expensive, and they are available only in specialized clinics. Our preliminary experience with liquid nitrogen cryoablation confirms that the procedure can be easily performed by any dermatologist and does not require great experience. Cryotherapy is less expensive than other procedures, and it is usually available during the visit in a common outpatient setting; thus, it is a candidate to be a valid alternative to the local surgery. The limitations of the study include the retrospective nature of the data, not allowing analysis of factors conditioning partial response and the number of patients. More studies are warranted to confirm the efficacy and consolidate protocols.

## Data availability statement

The original contributions presented in the study are included in the article/supplementary material, further inquiries can be directed to the corresponding author.

## Ethics statement

The studies involving human participants were reviewed and approved by Ethicis Committee of AOU Cagliari. The patients/participants provided their written informed consent to participate in this study.

## Author contributions

LA, CF, MD, JA, and SS: conception and design of the study. LA, CF, MD, JA, AT, SS, and AF: acquisition, analysis, and interpretation of data. LA, MD, AT, AF, and CF: writing of the article. LA: supervision. All authors have read and approved the final version of the manuscript.

## References

[1] Revuz J. Hidradenitis suppurativa. J Eur Acad Dermatol Venereol. (2009) 23:985–98. 10.1111/j.1468-3083.2009.03356.x 19682181

[2] Jemec GBE Heidenheim M Nielsen NH. The prevalence of hidradenitis suppurativa and its potential precursor lesions. J Am Acad Dermatol. (1996) 35:191–4. 10.1016/S0190-9622(96)90321-7 8708018

[3] Matusiak Ł Bieniek A Szepietowski JC. Psychophysical aspects of hidradenitis suppurativa. Acta Derm Venereol. (2010) 90:264–8. 10.2340/00015555-0866 20526543

[4] Esmann S Jemec GBE. Psychosocial impact of hidradenitis suppurativa: a qualitative study. Acta Derm Venereol. (2011) 91:328–32. 10.2340/00015555-1082 21394419

[5] Zouboulis CC Desai N Emtestam L. European S1 guideline for the treatment of hidradenitis suppurativa/acne inversa. J Eur Acad Dermatol Venereol. (2015) 29:619–44. 10.1111/jdv.12966 25640693

[6] Marzano AV Genovese G Casazza G. Evidence for a ‘window of opportunity' in hidradenitis suppurativa treated with adalimumab: a retrospective, real-life multicentre cohort study. Br J Dermatol. (2021) 184:133–40. 10.1111/bjd.18983 32119111

[7] Bong JL Shalders K Saihan E. Treatment of persistent painful nodules of hidradenitis suppurativa with cryotherapy. Clin Exp Dermatol. (2003) 28:241–4. 10.1046/j.1365-2230.2003.01238.x 12780702

[8] Pagliarello C Fabrizi G Feliciani C Di Nuzzo S. Cryoinsufflation for Hurley stage II hidradenitis suppurativa: a useful treatment option when systemic therapies should be avoided. JAMA Dermatol. (2014) 150:765–6. 10.1001/jamadermatol.2014.430 24806911

[9] Pagliarello C Fabrizi G di Nuzzo S. Cryoinsufflation for hidradenitis suppurativa: technical refinement to prevent complications. Dermatol Surg. (2016) 42:130–2. 10.1097/DSS.0000000000000572 26716713

[10] Molina-Leyva A Salvador-Rodriguez L Martinez-Lopez A Cuenca-Barrales C. Effectiveness, safety and tolerability of drainage and punch-trocar-assisted cryoinsufflation (cryopunch) in the treatment of inflammatory acute fluid collections in hidradenitis suppurativa patients. J Eur Acad Dermatol Venereol. (2019) 33:e221–3. 10.1111/jdv.15406 30811686

[11] Alikhan A Sayed C Alavi A. North American clinical management guidelines for hidradenitis suppurativa: a publication from the United States and Canadian hidradenitis suppurativa foundations: part I: diagnosis, evaluation, and the use of complementary and procedural management. J Am Acad Dermatol. (2019) 81:76–90. 10.1016/j.jaad.2019.02.067 30872156 PMC9131894

[12] Shah N. Hidradenitis suppurativa: a treatment challenge. Am Fam Phys. (2005) 72:1547–52. 16273821

[13] Goldburg SR Strober BE Payette MJ. Hidradenitis suppurativa: current and emerging treatments. J Am Acad Dermatol. (2020) 82:1061–82. 10.1016/j.jaad.2019.08.089 31604100

[14] Melendez Gonzalez MDM Sayed CJ. Surgery is an essential aspect of managing patients with hidradenitis suppurativa. J Am Acad Dermatol. (2020) 83:979–80. 10.1016/j.jaad.2020.03.008 32171813

[15] Fox L Csongradi C Aucamp M du Plessis J Gerber M. Treatment modalities for acne. Molecules. (2016) 21:1063. 10.3390/molecules21081063 27529209 PMC6273829

[16] Goette DK. Liquid nitrogen in the treatment of acne vulgaris: a comparative study. South Med J. (1973) 66:1131–2. 10.1097/00007611-197310000-00011 4270404

[17] Nazzaro G Passoni E Muratori S Moltrasio C Guanziroli E Barbareschi M. Comparison of clinical and sonographic scores in hidradenitis suppurativa and proposal of a novel ultrasound scoring system. Ital J Dermatol Venerol. (2021) 156:235–9. 10.23736/S2784-8671.18.06196-5 30298709

[18] Wortsman X. Strong validation of ultrasound as an imaging biomarker in hidradenitis suppurativa. Br J Dermatol. (2021) 184:591–2. 10.1111/bjd.19433 32869312

[19] Kuflik EG. Cryosurgery updated. J Am Acad Dermatol. (1994) 31:925–44. 10.1016/S0190-9622(94)70261-6 7962774

[20] Zimmerman EE Crawford P. Cutaneous cryosurgery. Am Fam Phys. (2012) 86:1118–24. 23316984

[21] Cook DK Georgouras K. Complications of cutaneous cryotherapy. Med J Aust. (1994) 161:210–3. 10.5694/j.1326-5377.1994.tb127385.x 8035726

[22] Ovadja ZN Jacobs W Zugaj M van der Horst CMAM Lapid O. Recurrence rates following excision of hidradenitis suppurativa: a systematic review and meta-analysis. Dermatol Surg. (2020) 46:e1–7. 10.1097/DSS.0000000000002403 32235148

[23] Hazen PG Hazen BP. Hidradenitis suppurativa: successful treatment using carbon dioxide laser excision and marsupialization. Dermatol Surg. (2010) 36:208–13. 10.1111/j.1524-4725.2009.01427.x 20039918

